# Investigating the effect of cochlear implant usage metrics on cortical auditory-evoked potential responses in adult recipients post-implantation

**DOI:** 10.3389/fnins.2024.1453274

**Published:** 2024-11-20

**Authors:** Caris Bogdanov, Helen Goulios, Wilhelmina H. A. M. Mulders, Dayse Tavora-Vieira

**Affiliations:** ^1^School of Human Sciences, University of Western Australia, Perth, WA, Australia; ^2^Department of Audiology, Fiona Stanley Fremantle Hospitals Group, Perth, WA, Australia; ^3^Division of Surgery, Medical School, University of Western Australia, Perth, WA, Australia; ^4^School of Allied Health, Faculty of Health Sciences, Curtin University, Perth, WA, Australia

**Keywords:** cochlear implant fitting, cortical auditory-evoked potentials, objective measures, sensorineural hearing loss, P1-N1-P2 complex, personalized hearing rehabilitation

## Abstract

**Introduction:**

This study examines the effect of cochlear implant (CI) device usage metrics on post-operative outcomes in unilateral CI recipients. The primary objective is to investigate the relationship between CI usage frequency (average daily CI use) and duration (total years of CI use) on electrically evoked cortical auditory-evoked potential (eCAEP) response peak latency (ms) and amplitude (μV).

**Methods:**

Adult CI users (*n* = 41) who previously exhibited absent acoustically evoked CAEP responses participated in the study. The peak latency and amplitude of eCAEP P1-N1-P2 responses were recorded, when present for the apical, medial, and basal test electrode contacts. CI duration was defined as the number of years between the date of CI activation and date that eCAEP testing was performed. CI usage frequency was defined as the average number of hours per day of audio processor use, which was recorded using the CI programming software.

**Results:**

Overall, 27 participants (65.85%) exhibited detectable eCAEP responses across one or more electrode contacts. Among these, 18 participants (43.9%) elicited eCAEP responses at all three electrode contacts, while 7 (17.07%) showed responses at two contacts, and 2 (4.88%) at one contact. For the remaining 14 participants (34.15%), eCAEP responses were either absent or undetectable. CI usage frequency (average daily CI use [hours/day]) was captured for 32 (78%) of the participants (median 10.35 h/day, range 0.2–16 h/day). Participants with present eCAEP responses for the basal electrode (*n* = 14) showed significantly higher CI usage frequency (11.8 h/day, *p* = 0.026) compared to those with non-detectable responses (6.25 h/day). An association was found between higher CI usage frequency and reduced N1 (*p* = 0.002), P2 (*p* = 0.0037) and P1-N1 inter-peak (*p* = 0.015) response latency (ms). While CI duration (total CI use [years]) did not differ significantly between groups based on the presence of eCAEP responses, an association was found between greater CI duration and increased eCAEP response amplitude (μV) for the P2 (*p* = 0.008) and N1-P2 peak-peak (*p* = 0.009) response components.

**Discussion:**

Additionally, most (65.85%) participants who previously exhibited absent acoustic CAEP responses developed eCAEP responses after consistent CI use and increased CI experience. These findings may suggest a potential for cortical plasticity and adaptation with consistent CI use over time. Recognizing the impact of device usage metrics on neural responses post-implantation enhances our understanding of the importance of consistent daily CI use. Overall, these findings contribute to addressing the variability among CI users, improving post-operative outcomes and advancing the standard of personalized care in auditory rehabilitation.

## Introduction

Hearing loss poses a significant global health challenge, impacting communication, social engagement, and overall quality of life (Punch et al., 2019; Harithasan et al., 2020). For people with severe to profound sensorineural hearing loss, a cochlear implant (CI) can restore access to auditory information through direct electrical stimulation of the auditory nerve, and is the gold standard for hearing rehabilitation in this patient population (Naples and Ruckenstein, 2020); (Buchman et al., 2020). Despite this, unexplained variability in performance among adult CI users post-implantation remains a challenge (Moberly et al., 2016; Ma et al., 2022).

While many CI users achieve excellent outcomes, some show limited or no hearing improvement and continue to face significant challenges, especially understanding speech in noise (Fu and Nogaki, 2005; Nelson et al., 2003; Stickney et al., 2004; Cullington and Zeng, 2008; Holden et al., 2013). Understanding the underlying factors contributing to this variability is crucial to improving rehabilitation outcomes for all CI recipients. The sources of variability involve a range of factors, including demographics (e.g., age, age at implantation, duration of deafness, and etiology of hearing loss), surgical placement (electrode array insertion depth and location), inner ear malformations, neural health (involving survival of spiral ganglion neurons and cortical neural plasticity), and higher-order cognitive functions (e.g., verbal working memory, attention, executive function, and learning abilities) (Blamey et al., 2013; Blamey et al., 1996; Simon et al., 2020; Zhao et al., 2020; Holden et al., 2013; Finley et al., 2008). Additionally, the likelihood of CI success post-operatively may be limited by discrepancies in clinical mapping and programming parameters of the external audio processor, consequently impacting stimulation specificity and efficacy (Távora-Vieira and Marino, 2019; Martins and Goffi-Gomez, 2021).

Traditionally, CI mapping and programming rely on the psychoacoustic responses of the recipient to determine device parameters (electrical thresholds [Ts], and most comfortable loudness levels [MCLs]). Recent research has highlighted the limitations associated with the subjective nature of this approach, which may be susceptible to non-auditory influences, such as the CI recipient’s cognitive status or the presence of tinnitus, potentially leading to conservative CI mapping that prioritizes comfort over adequate sound stimulation (Távora-Vieira and Marino, 2019). Hence, the risk of under-or over-stimulation is increased when programming devices for recipients who cannot provide consistent feedback or reliably report loudness sensation, such as infants, young children, people with pre-lingual deafness, or people with cognitive impairment (Vargas et al., 2013). Therefore, while subjective programming is effective for most CI users, it does not consistently produce optimal electrical stimulation for speech perception for all users, thereby limiting the potential for CI users to derive the maximum benefit from their CI.

Using objective measures to program CIs is a potential solution to these limitations. Specifically, the use of Cortical Auditory-Evoked Potential (CAEP) responses provides an objective, physiological measure to verify the behavioral responses used in CI programming (Kosaner et al., 2018; Távora-Vieira et al., 2022a; Távora-Vieira et al., 2022b; Távora-Vieira et al., 2018). Studies have demonstrated the effectiveness of using acoustically-evoked CAEP (aCAEP) responses to verify hearing aid fittings and, more recently, their use in the CI context suggests a strong correlation between the presence of aCAEP responses and improved auditory outcomes (Purdy et al., 2001; Korczak et al., 2005; Glista et al., 2012; Golding et al., 2007; Alvarenga et al., 2012; Chang et al., 2012; Carter et al., 2013; Kelly et al., 2005; Visram et al., 2015; Sharma et al., 2005).

Távora-Vieira et al. (2018) validated the clinical use of aCAEP measures as an objective CI verification tool to confirm the audibility of sounds across different frequencies at the level of the auditory cortex. Recent studies demonstrated a significant improvement in speech perception scores in CI users who underwent aCAEP-guided CI map adjustments based on the presence of the P1-N1-P2 complex (Távora-Vieira et al., 2022b; Távora-Vieira et al., 2022a), and this was irrespective of specific patient or device factors (Bogdanov et al., 2023). While these findings reveal the benefit of objectively verifying CI mapping using aCAEP measures to improve speech perception outcomes, the use of aCAEPs relies on an automated method to determine the presence or absence of the P1-N1-P2 complex in response to acoustic stimulation. Therefore, electrically-evoked cortical auditory-evoked potential (eCAEP) responses have recently been used to overcome this limitation and improve our understanding of cortical activation in response to CI stimulation using specific P1-N1-P2 complex response metrics (i.e., amplitude and latency).

The use of eCAEPs provides an objective measure for direct stimulation of specific CI electrode contacts, avoiding the acoustic-to-electric conversion inherent in aCAEPs. This direct stimulation method allows precise information specific to the function of each electrode contact to be captured. This could enable the creation of a more accurate and personalized approach to CI programming (Kelly et al., 2005; Távora-Vieira et al., 2018; Távora-Vieira et al., 2022b; Távora-Vieira et al., 2022a). In contrast to the aCAEP method, additional benefits of using eCAEPs include their independence from external auditory stimuli, the lack of requirement for a sound-proof environment, and the ability to target specific electrode contacts for stimulation, thus providing a more detailed profile of the CI map to verify MCLs. Thus, eCAEPs may be a beneficial objective measure for fitting some CI users; providing a method to streamline the fitting process by reducing the time and cognitive load required of recipients during the CI mapping procedure.

Despite the relative novelty of this technique, a comprehensive validation of the eCAEP method has been conducted, which successfully compared aCAEP and eCAEP recordings in CI users, yielding clear and reliable acoustic and electrical P1–N1–P2 responses that exhibited strong correlation (Távora-Vieira et al., 2021). Additionally, Tavora-Vieira and Ffoulkes (2023) demonstrated that eCAEPs can be readily elicited at stimulation levels corresponding to MCL, providing a reliable method to assess sound perception in adult CI users, which is unaffected by age, duration of deafness, or hearing loss laterality. However, these studies have not provided any insight into those CI users who did not obtain cortical responses evoked by electrical stimulation.

In addition to the research investigating the impact of auditory stimulation and optimal CI mapping post-operatively, numerous studies have evaluated the relationship between the duration of CI experience and extent of daily device usage to predict CI success and speech recognition outcomes (Lindquist et al., 2023; Holder and Gifford, 2021; Holder et al., 2020; DeFreese et al., 2023; Ma et al., 2022). While the importance of long-term CI use for improvement in outcomes over time is well-established (Ma et al., 2022; Spivak and Waltzman, 1990; Buss et al., 2008), more recent studies have demonstrated that increased daily CI use results in improved auditory perception and speech recognition scores for adults, even in those with extensive CI experience (Holder and Gifford, 2021).

In light of these findings, the present study seeks to expand upon prior research by examining the effects of usage metrics, CI usage frequency (average daily CI use) and duration (total years of CI use) on post-operative outcomes in unilateral CI recipients. The primary objective is to investigate the relationship between device usage metrics, specifically CI usage frequency and duration on eCAEP response peak latency (ms) and amplitude (μV). Additionally, this study aims to further our understanding of the association between eCAEP responses and factors related to CI outcomes and inter-patient variability, such as age, sex, type, and etiology of hearing loss, as well as device-specific variables. We hypothesize that increased frequency and duration of device use facilitates the development of robust cortical responses in adult CI users, thereby reinforcing the benefit of using objective measures to monitor and optimize outcomes in adult CI recipients.

## Materials and methods

### Participants

Participants were recruited from the audiology department at a tertiary hospital in Western Australia. To be included in the study, participants had to: be adult (18–90 years of age) unilateral CI users with acquired post-lingual bilateral or single-sided deafness; have undergone CI surgery at least 12 months prior to testing; and have previously exhibited absent acoustic CAEP responses, despite CI mapping adjustments.

### eCAEP response recording

**Figure 1:**
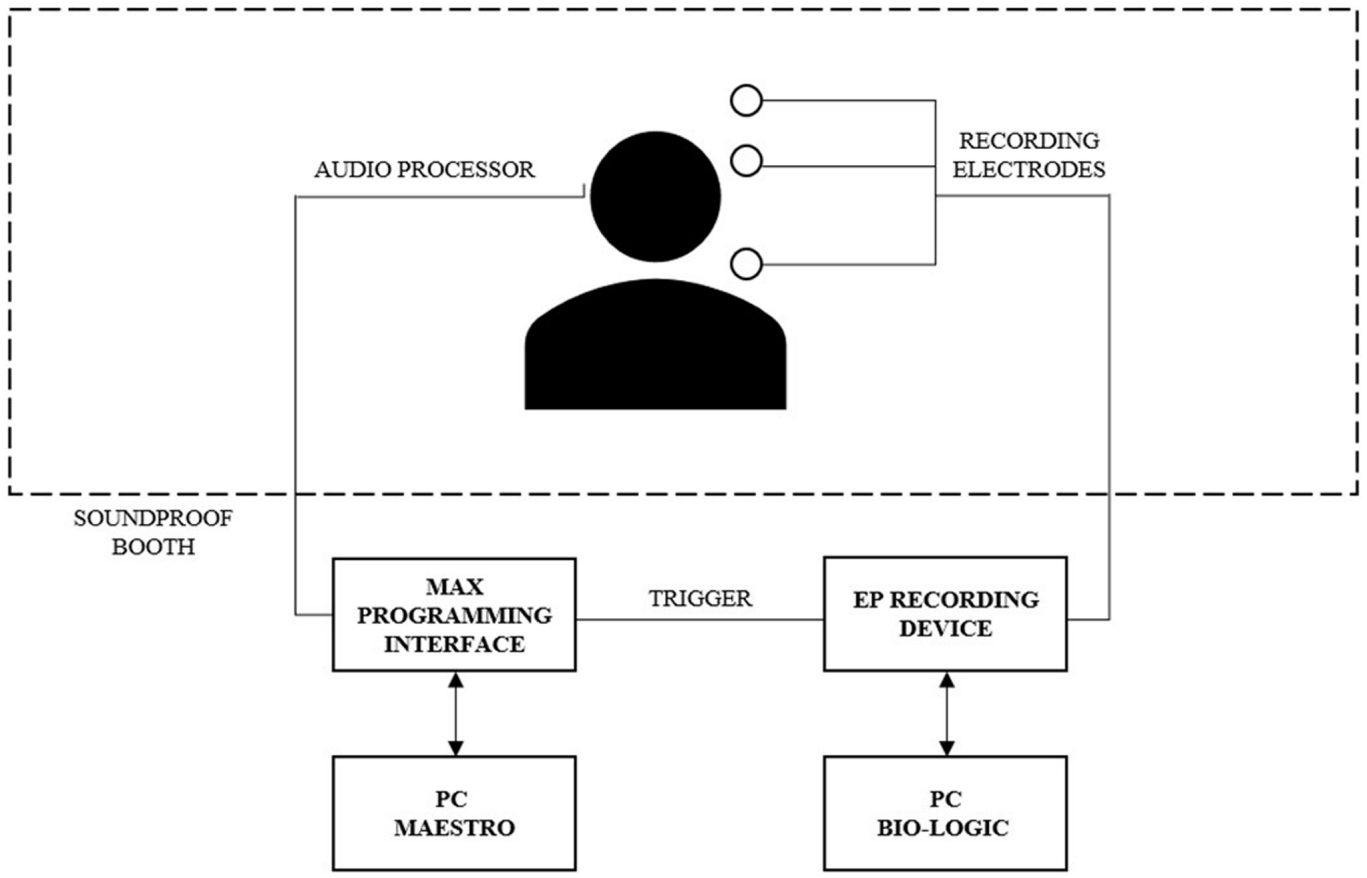
Schematic of eCAEP recording apparatus set-up.

Participant eCAEP responses were elicited by direct electrical stimulation using the MAESTRO 9.0.1 (MED-EL, Innsbruck, Austria) eABR module and recorded via the Bio-logic Navigator Pro (Natus, Pleasanton, CA, USA). Signal synchronization was achieved using a trigger cable connecting the CI programming interface and the Bio-Logic recording equipment (Figure 1). The eABR module presented the electrical burst stimuli at a rate of 0.9 Hz and with a duration of 70 ms. Stimuli were composed of a series of biphasic symmetric alternating pulses, each with a phase duration of 40 μs, presented at a stimulation rate of 1 kHz. Participant eCAEP responses were recorded from 60 ms pre-stimulus (baseline) to 500 ms post-stimulus onset, with a minimum of 100 epochs per stimulus. This protocol ensures the electrical artifacts generated by the CI are excluded and the entire response is captured, including the P2 peak. To obtain eCAEP responses, stimuli were presented at participant MCL levels for three electrode contacts: electrode 1 (apical), 6 (medial), and 11 (basal). The eCAEP responses were recorded non-invasively using a three-electrode montage, consisting of a non-inverting, inverting, and ground electrode, placed on the vertex (Cz), mastoid contralateral to the CI, and forehead, respectively. Electrode impedance was maintained below 5 kΩ and the impedance differences between the electrodes were maintained at <2 kΩ. Participant responses were recorded until 100 valid averages were obtained. An analog bandpass (0.3–100 Hz) filtered the signal, and a notch filter (60 Hz) was applied if required. Participants were instructed to stay relaxed and minimize muscle contraction during the recording session Alertness was monitored by the supervising clinician. Data reliability was confirmed by reproducibility of the recording.

### P1-N1-P2 complex

The eCAEP data were exported in txt format for further analysis using Python (Python Software Foundation, version 3.9). To validate the accurate identification of present and absent eCAEP responses, three experienced audiologists in electrophysiology independently visually inspected the waveform morphology of the P1, N1, and P2 components. The eCAEP responses were confirmed by unanimous agreement on the presence of the P1-N1-P2 complex. All three audiologists identified the positive (P1, P2) and negative (N1) peaks, which were manually marked at the peak apex to calculate the response amplitude (μV), determined from peak-to-peak, and latency (ms), measured from stimulus onset to peak onset. The eCAEP response amplitude (μV) and latency (ms) was derived from the average values recorded by each audiologist for the P1, N1, and P2 components identified within the expected range.

### Device use metrics

Duration of CI use was defined as the number of years elapsed between the date of CI activation and date that eCAEP testing was performed. The average number of hours per day of audio processor use was recorded using the CI programming software. The datalogging value was captured at the time of eCAEP test and was calculated based on the usage time since the previous appointment. CI users with devices, e.g., RONDO series or OPUS 2, that do not support datalogging were excluded from this analysis.

### Statistical analysis

All statistical analyses were conducted using R statistics and R Studio software (RStudio, 2020). Normality (via the Shapiro–Wilk test) and equal variance (via the F test) were determined on the basis of which parametric or non-parametric data analysis was conducted. The Mann–Whitney U Test was used for comparisons between quantitative variables stratified by categorical variables, and the Kruskal-Wallis H test was used for comparisons between >2 variables. The Chi-Square (χ^2^) test of independence was used for comparisons between categorical variables. Spearman rank correlation was used to estimate correlations between quantitative variables. Linear regression model analysis was performed for eCAEP response latency (ms) and amplitude (μV) to estimate the effect of the average daily CI usage frequency and duration of CI use and modeled according to the test electrode contact. To control for the risk of type 1 error, we implemented the Benjamini-Hochberg method to adjust the significance level for multiple comparisons.

## Results

### Participants

A total of 41 adult CI users (23 males; 18 females) with bilateral (*n* = 29) and single-sided deafness (*n* = 12) participated in this study. Individual demographic, aetiological, and clinical data are summarized in Table 1. All participants had absent acoustic CAEP responses post-operatively, measured and recorded using the HEARLab system, as per the protocol described by Távora-Vieira et al. (2022a) and Távora-Vieira et al. (2022b).

**Table 1:** Summary of participant demographics, hearing loss onset (nature and etiology), device information and stimulation parameters at the time of eCAEP testing.

Demographic data				Device data				eCAEP data						
Gender	Onset nature	Etiology	Age at implant (yrs)	Implant ear	Implant	Array	Audio processor	Age at test (yrs)	CI duration (yrs)	CI frequency (hrs/day)	STIM mode	MCL (qu)		
												Apical	Medial	Basal
F	Gradual	VASCULAR	81	R	SYNCHRONY 2	FLEX28	SONNET 2	82	1.1	10.3	BIPHASIC	12.74	16.42	17.73
M	Gradual	NIHL	79	R	SYNCHRONY 2	FLEX28	SONNET 2	82	2.2	4.9	BIPHASIC	20.60	30.90	22.25
F	Sudden	ISSNHL	87	R	SYNCHRONY 2	FLEX28	SONNET 2	89	2.1	6.5	BIPHASIC	16.52	19.51	18.33
M	Gradual	NIHL	80	L	SYNCHRONY 2	FLEX24	SONNET 2	80	1	11.6	BIPHASIC	23.50	30.22	29.95
M	Gradual	SNHL	85	L	SYNCHRONY 2	FLEX28	SONNET 2	87	2.6	16	BIPHASIC	24.54	30.22	26.78
M	Sudden	VIRUS	66	R	SYNCHRONY 2	STANDARD	SONNET 2	70	4	13	BIPHASIC	14.44	14.99	17.06
M	Gradual	SNHL	72	L	SYNCHRONY	FLEX28	SONNET 2	78	6.5	15.7	TRIPHASIC	19.01	23.06	21.14
F	Sudden	TRAUMA	65	L	SYNCHRONY 2	FLEXSOFT	RONDO 2	71	5.8	14	BIPHASIC	21.19	24.70	18.76
F	Gradual	SNHL	73	L	SYNCHRONY	FLEX28	SONNET	78	5	12.4	BIPHASIC	15.55	21.49	21.28
F	Gradual	UNKNOWN	73	R	SYNCHRONY 2	FLEX28	RONDO 3	74	1.4	4.8	BIPHASIC	22.21	27.67	23.48
F	Gradual	UNKNOWN	61	R	SYNCHRONY	FLEX28	SONNET	68	7.1	12.8	BIPHASIC	28.18	27.74	19.37
M	Gradual	OTOSCLEROSIS	68	L	SYNCHRONY 2	FLEX28	SONNET 2	70	2.5	15.2	BIPHASIC	15.22	16.42	18.23
F	Gradual	VIRUS	66	L	SONATAti100	STANDARD	SONNET 2	80	13.9	0.2	BIPHASIC	16.70	24.79	20.50
M	Gradual	MD	70	R	SYNCHRONY 2	FLEX28	SONNET 2	73	3	13	BIPHASIC	36.83	29.03	27.43
F	Sudden	ISSNHL	73	L	SYNCHRONY 2	FLEX28	SONNET	78	4.9	10	BIPHASIC	21.75	24.18	30.26
F	Sudden	OTOSCLEROSIS	70	R	SYNCHRONY 2	FLEX28	SONNET 2	73	3.7	14.8	BIPHASIC	25.19	37.17	29.02
M	Gradual	VIRUS	73	L	SYNCHRONY	FLEX28	RONDO 3	80	7.1	1.7	TRIPHASIC	31.13	32.75	36.98
M	Sudden	ISSNHL	70	L	SYNCHRONY	FLEX28	SONNET	74	4.6		BIPHASIC	16.78	24.59	24.20
M	Gradual	NIHL	75	L	SYNCHRONY 2	FLEX28	SONNET 2	76	1	9	BIPHASIC	17.56	19.15	15.83
F	Gradual	SNHL	86	R	SYNCHRONY 2	FLEX28	SONNET 2	89	2.6	10.4	BIPHASIC	15.56	17.24	15.42
M	Sudden	TRAUMA	57	R	SYNCHRONY 2	FLEX28	SONNET 2	58	1.11	3.6	BIPHASIC	24.78	29.13	25.50
M	Sudden	CONGENITAL	60	L	SYNCHRONY 2	FLEX28	SONNET 2	63	2.5	2.6	TRIPHASIC	16.90	17.34	16.18
F	Sudden	CONGENITAL	49	L	CONCERTO	FLEX28	SONNET 2	58	8.7	11.5	BIPHASIC	10.09	13.49	17.30
M	Gradual	MD	58	R	SYNCHRONY 2	FLEX28	SONNET 2	63	4.9	15.8	TRIPHASIC	18.46	19.79	18.73
F	Gradual	MEP	62	L	SYNCHRONY 2	FLEX26	SONNET 2	63	1.4	14.6	BIPHASIC	24.78	29.01	24.60
M	Sudden	ISSNHL	59	R	SYNCHRONY 2	FLEX28	SONNET 2	61	2.11	8.8	BIPHASIC	18.87	22.37	16.42
F	Gradual	SNHL	75	R	SONATAti100	FLEXSOFT	OPUS 2	88	13.11		BIPHASIC	22.76	27.86	19.03
M	Gradual	CONGENITAL	19	L	CONCERTO	STANDARD	RONDO 3	27	8.7	12	BIPHASIC			
F	Sudden	ISSNHL	41	L	SYNCHRONY 2	FLEX28	RONDO 3	44	2.6	5.9	TRIPHASIC	20.70	22.93	25.07
M	Sudden	ISSNHL	63	L	SYNCHRONY 2	FLEX26	RONDO 2	63	1		BIPHASIC	10.14	12.24	14.87
M	Gradual	NIHL	44	R	SYNCHRONY	FLEX28	SONNET	47	3	13.2	TRIPHASIC	33.99	34.55	30.10
F	Sudden	ISSNHL	31	R	SYNCHRONY 2	FLEX28	RONDO 2	32	1		BIPHASIC	23.45	26.03	23.73
F	Sudden	ISSNHL	35	R	SYNCHRONY 2	FLEX28	SONNET 2	37	2	1.8	TRIPHASIC	16.60	20.17	18.94
M	Sudden	MEP	68	R	CONCERTO	FLEX24	RONDO	76	7.5		BIPHASIC	18.57	22.88	22.96
F	Sudden	ISSNHL	76	R	SYNCHRONY 2	FLEX24	SONNET	79	2.7	7.95	BIPHASIC	23.27	27.99	19.94
M	Sudden	NEUROPATHY	42	R	SYNCHRONY 2	FLEX28	SONNET 2	43	1	6	BIPHASIC	19.15	23.49	18.51
M	Gradual	SNHL	69	R	SYNCHRONY	FLEX28	RONDO 3	77	7.7		TRIPHASIC	16.20	16.54	16.91
M	Gradual	SNHL	82	R	SYNCHRONY 2	FLEX28	RONDO 3	85	2.11	2.1	BIPHASIC	25.25	29.24	22.95
M	Gradual	SNHL	87	R	SYNCHRONY 2	FLEX28	SONNET 2	90	2.8	5.7	TRIPHASIC	26.54	29.52	29.89
M	Sudden	TRAUMA	41	R	SONATAti100	STANDARD	RONDO	54	13.5		TRIPHASIC	20.14	23.85	25.62
F	Sudden	MEP	39	L	SYNCHRONY 2	FLEX28	SONNET 2	43	3.4	10.5	TRIPHASIC	19.10	19.10	19.10

### eCAEP response

The eCAEP P1-N1-P2 responses, including both peak latency (ms) and amplitude (μV) were recorded for the apical, medial, and basal test electrode contacts. Participants were categorized based on whether they exhibited the P1-N1-P2 eCAEP response at these electrode contact positions. Detectable eCAEP responses were exhibited by 27 participants (65.85%) across one or more electrode contacts. Among these, 18 participants (43.9%) elicited eCAEP responses at all three electrode contacts, while 7 (17.07%) showed responses at two contacts, and 2 (4.88%) at one contact. For the remaining 14 participants (34.15%), eCAEP responses were either absent or undetectable. Results for individual electrode contact positions can be seen in Table 2.

**Table 2:** Median (IQR) P1-N1-P2 eCAEP response peak latency (ms) and amplitude (μv) for apical, medial, and basal electrode contact positions.

Electrode contact									
		Apical		Medial		Basal		Statistic	*p*-value
Peak latency (ms)									
P1	*n*	26		26		19			
	*median (IQR)*	36.5	(30.95–52)	37.25	(33.4–44.73)	35.3	(30.82–49.8)	0.177	0.916
N1	*n*	26		26		19			
	*median (IQR)*	87.6	(85.58–97.17)	86.8	(81–93.22)	84	(77.8–90.15)	3.853	0.146
P2	*n*	25		26		19			
	*median (IQR)*	202	(176–209.1)	186.1	(175.1–219.2)	178.6	(154.4–203.1)	1.569	0.456
P1-N1	*n*	26		26		19			
	*median (IQR)*	49	(40.42–60.98)	49.8	(43.8–55.85)	46.4	(42–54.2)	0.77	0.68
N1-P2	*n*	25		26		19			
	*median (IQR)*	107	(84.3–125.8)	104.2	(90.3–125.8)	94.3	(77.45–112.9)	1.08	0.583
Peak amplitude (μV)									
P1	*n*	26		26		19			
	*median (IQR)*	0.67	(−0.32–1.2)	0.49	(−1.83–1.49)	0.76	(−0.96–2.09)	0.776	0.678
N1	*n*	26		26		19			
	*median (IQR)*	−4.53	(−6.43 – −2.34)	−5.2	(−6.34 – −2.36)	−4.2	(−5.07 – −2.93)	0.267	0.875
P2	*n*	25		26		19			
	*median (IQR)*	2.7	(1.28–4.30)	2.245	(1.39–3.73)	2.47	(0.59–4.03)	0.098	0.952
P1-N1	*n*	26		26		18			
	*median (IQR)*	4.53	(2.93–6.96)	4.65	(2.84–5.79)	4.34	(3.45–6.75)	0.034	0.983
N1-P2	*n*	25		26		18			
	*median (IQR)*	6.6	(4.64–9.90)	7.84	(4.28–10.23)	6.94	(4.38–8.79)	0.268	0.875

### Participant age

**Figure 2:**
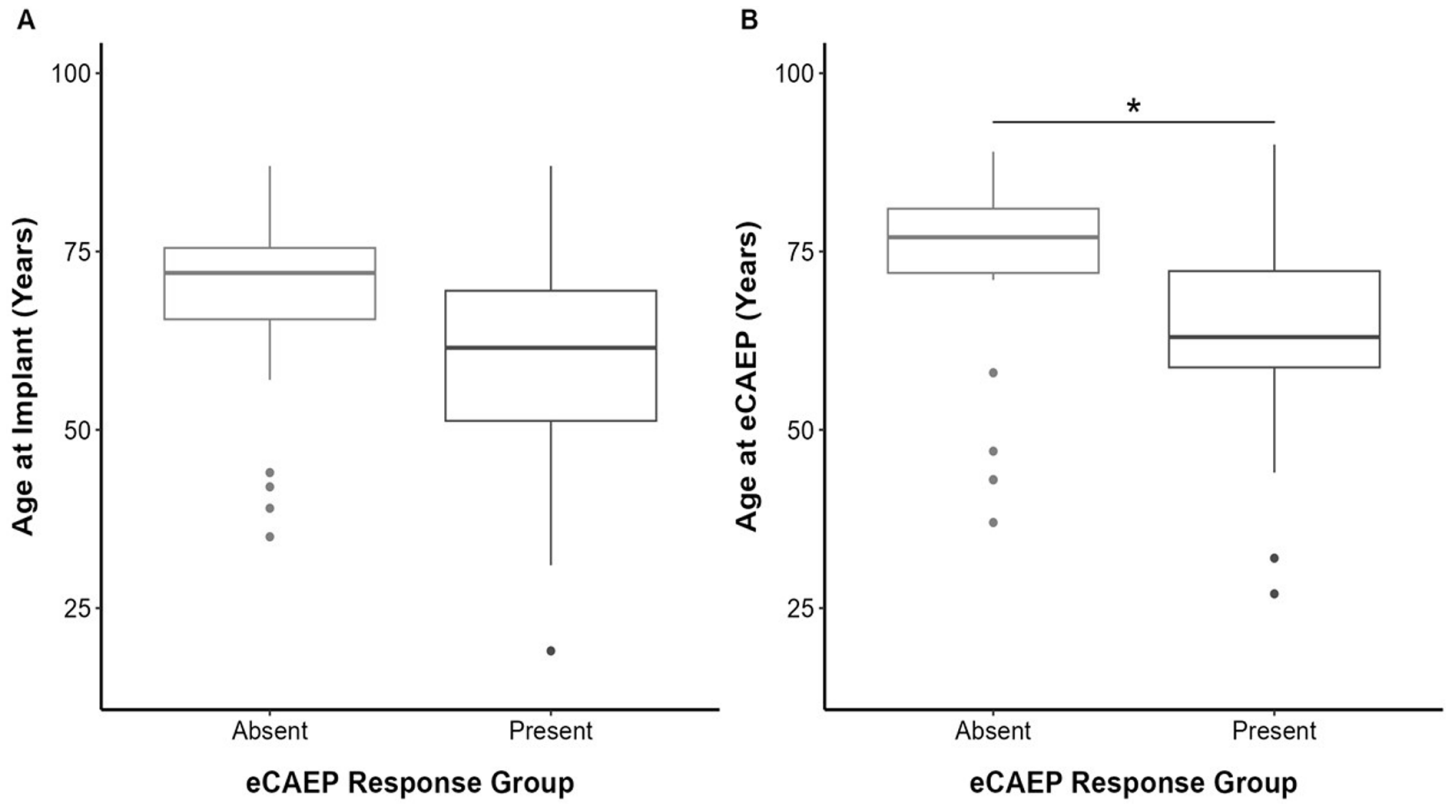
Boxplot depicts median (central line), interquartile range (box) and whiskers extending to show the full range of participant age (years) at **(A)** implantation and **(B)** testing compared between absent and present eCAEP response groups. Statistical significance (*) is indicated where applicable. Panel **(A)** shows no significant difference in age at implantation between participants with absent (*n* = 23, 72 years [65.5–75.5 years]) or present (*n* = 18, 61.5 years [51.25–69.5 years]) eCAEP responses. Panel **(B)** shows age at the time of testing was significantly higher (*U* = 286.5, *p* = 0.038) in participants with absent eCAEP responses (*n* = 23, 77 years [72–81 years]), compared to those with present eCAEP responses (*n* = 18, 63 years [58.75–72.25 years]).

The median participant age was 73 years (range 27–90 years) at eCAEP testing and 68 years (range 19–87 years) at implantation. Comparisons between eCAEP response groups showed that participant age at testing was significantly greater (*U* = 286.5, *p* = 0.038) in the participants with non-detectable response(s) (median 70 years, range 32–90 years) than those with present responses (median 64 years, range 27–78 years) for all electrode contacts (Figure 2). No association was observed between eCAEP response groups and age at implantation (*U* = 278, *p* = 0.064; Figure 2). Furthermore, no association was observed between participant age at testing or implantation when stratified by electrode contact position (apical, medial, and basal), as detailed in Table 3.

**Table 3:** Statistical analyses using Mann–Whitney U test to compare participant age (years) at the time of eCAEP testing and at implantation between eCAEP response groups for the apical, medial and basal electrode contact positions (data presented using total count [n] and median [IQR]).

Electrode contact		Response group			
		Present	Absent	Statistic	*p*-value
Age at eCAEP (Years)					
Apical	*n*	26	15	250	0.14
	*median (IQR)*	70 (61.5–77.75)	78 (64.5–86)		
Medial	*n*	26	15	250.5	0.136
	*median (IQR)*	70 (61.5–76.75)	78 (64.5–82.5)		
Basal	*n*	19	22	272.5	0.099
	*median (IQR)*	63 (59.5–75.5)	76.5 (71.5–80)		
Age at implant (Years)					
Apical	*n*	26	15	234.5	0.291
	*median (IQR)*	67 (58.25–73)	72 (61–79)		
Medial	*N*	26	15	225	0.424
	*median (IQR)*	67 (58.25–73)	72 (61–75.5)		
Basal	*n*	19	22	263	0.162
	*median (IQR)*	62 (53.5–71.5)	71 (65.25–75)		

### Device use: CI usage frequency and CI duration

**Figure 3:**
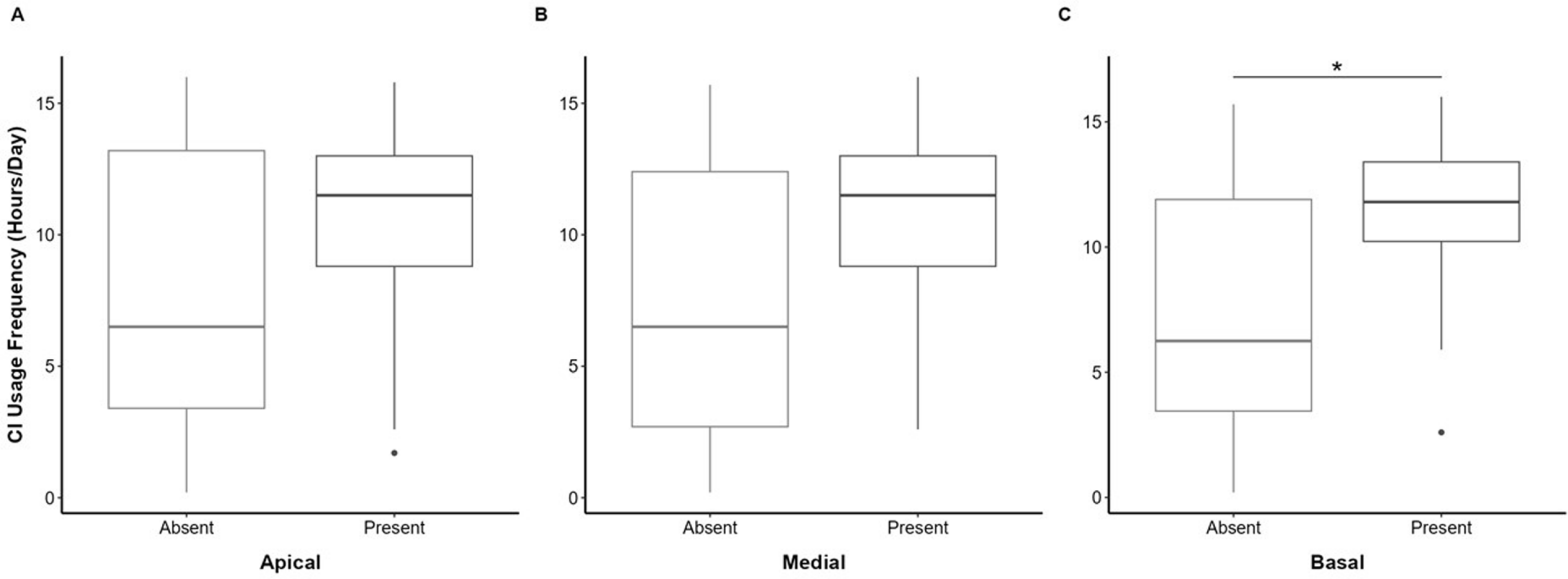
Boxplot depicts median (central line), interquartile range (box) and whiskers extending to show the full range of daily CI usage frequency (hours/day [h/d]) compared between absent and present eCAEP response groups categorized by **(A)** apical, **(B)** medial, and **(C)** basal test electrode contacts. Statistical significance (*) is indicated where applicable. Panel **(C)** shows that CI frequency was significantly higher (*U* = 79, *p* = 0.026) in participants with present eCAEP responses (*n* = 16, 11.8 h/d [10.22–13.4 h/d]), compared to those with absent eCAEP responses (*n* = 18, 6.25 h/d [3.45–11.9 h/d]) for the basal electrode contact. CI frequency was not significantly different (A; *U* = 103 *p* = 0.242, B; *U* = 86, *p* = 0.076) between participant groups differentiated by the presence of eCAEP responses for the **(A)** apical (absent; *n* = 13, 6.5 h/d [3.4–13.2 h/d], present; *n* = 21, 11.5 h/d [8.8–13 h/d]) or **(B)** medial (absent; *n* = 13, 6.5 h/d [2.7–12.4 h/d], present; *n* = 21, 11.5 h/d [8.8–13 h/d]) electrode contacts, respectively.

**Figure 4:**
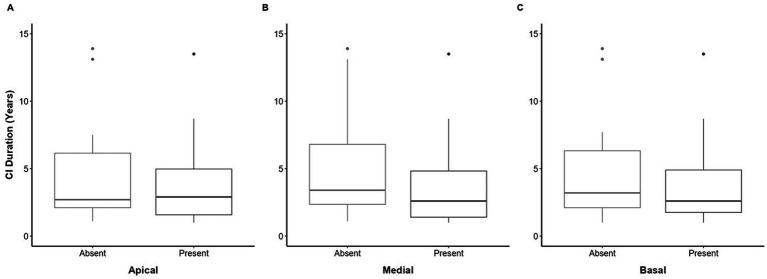
Boxplot depicts median (central line), interquartile range (box) and whiskers extending to show the full range of CI duration (total CI experience [years]) compared between absent and present eCAEP response groups categorized by **(A)** apical, **(B)** medial, and **(C)** basal test electrode contacts. CI duration was not significantly different (A; *U* = 210, *p* = 0.69, B; *U* = 253, *p* = 0.114, C; *U* = 240.5, *p* = 0.41) between participant groups differentiated by the presence of eCAEP responses for the **(A)** apical (absent; *n* = 15, 2 years [2–5.5 years], present; *n* = 26, 2.5 years [1.15–4.75 years]), **(B)** medial (absent; *n* = 15, 3 years [2–6.5 years], present; *n* = 26, 2 years [1–4 years]) or **(C)** basal (absent; *n* = 22, 3 years [2–5.75 years], present; *n* = 19, 2 years [1.5–4 years]) electrode contacts, respectively.

The CI usage frequency (average daily CI use [hours/day, h/d]) was captured for 32 of the 41 (78%) participants. The median CI usage frequency was 10.35 h (range 0.2–16 h/d). Analysis revealed that participants with detectable eCAEP responses (*n* = 16) demonstrated a median CI usage frequency (11.8 h/d, IQR 10.22–13.4 h/d), which was significantly higher (*U* = 79, *p* = 0.026) than the CI usage frequency (6.25 h/d, IQR 3.45–11.9 h/d) of those non-detectable responses (*n* = 18) for the basal electrode, but no significant difference in CI usage frequency was observed between eCAEP response groups for the apical and medial electrode contacts (Figure 3). In addition, no correlation between participant age at eCAEP testing (years) and CI usage frequency (h/d) was found for the total cohort (*r*(32) = 0.63, *p* = 0.527) or within the absent (*r*(17) = 0.88, *p* = 0.389) and present (*r*(13) = 0.46, *p* = 0.652) eCAEP response groups. The median CI duration (total years of CI use) was 2 years (range 2–5 years). Overall, CI duration was not significantly different between participant groups differentiated by the presence of eCAEP responses for the apical, medial or basal electrode contacts, as detailed in Figure 4.

### Demographic and device factors

No associations were observed between the presence of eCAEP responses and device factors, including the type of implant [χ^2^ (1, *N* = 41) = 3.68, *p* = 0.298], electrode array [χ^2^ (4, *N* = 41) = 6.013, *p* = 0.198], audio processor type [χ^2^ (5, *N* = 41) = 2.132, *p* = 0.831] or implantation side [χ^2^ (1, *N* = 41) = 0.144, *p* = 0.705]. Furthermore, analysis comparing participant sex [χ^2^ (1, *N* = 41) = 0.065, *p* = 0.799], nature [χ^2^ (1, *N* = 41) = 0.01, *p* = 0.92] and etiology of hearing loss [χ^2^ (11, *N* = 41) = 13.19, *p* = 0.281] showed no association between groups differentiated by the presence of eCAEP responses.

### Stimulus parameters: method, electrode contact position, and stimulation level

The stimulus parameters, including the method (biphasic or triphasic) and electrode contact position, matched the participant’s preferred settings to maintain comfort. If a participant had a disabled electrode contact (due to non-auditory stimulation or extracochlear placement), the next consecutive electrode was used to present the stimulus. No associations were found for the method of stimulation [χ^2^ (1, *N* = 41) = 0.015, *p* = 0.904], between participants using biphasic or triphasic settings and the presence of eCAEP responses. Similarly, no statistically significant correlations were observed between the electrode contact position; apical [χ^2^ (2, *N* = 41) = 4.78, *p* = 0.091], medial [χ^2^ (1, *N* = 41) = 0.609, *p* = 0.435] or basal [χ^2^ (2, *N* = 41) = 1.32, *p* = 0.516], used to deliver the stimulus and elicit the eCAEP response. Overall, the median stimulation level (qu) for the apical (19.64 qu, IQR 16.68–23.76 qu), medial (54.02 qu, IQR 19.42–28.55 qu) and basal (20.82 qu, IQR 18.30–25.18 qu) electrode contact positions showed no significant association between the presence of eCAEP responses for the apical (*U* = 210, *p* = 0.692), medial (*U* = 256, *p* = 0.101) and basal electrodes (*U* = 204, *p* = 0.827), respectively.

### eCAEP response waveform components: latency (ms) and amplitude (μV)

**Figure 5:**
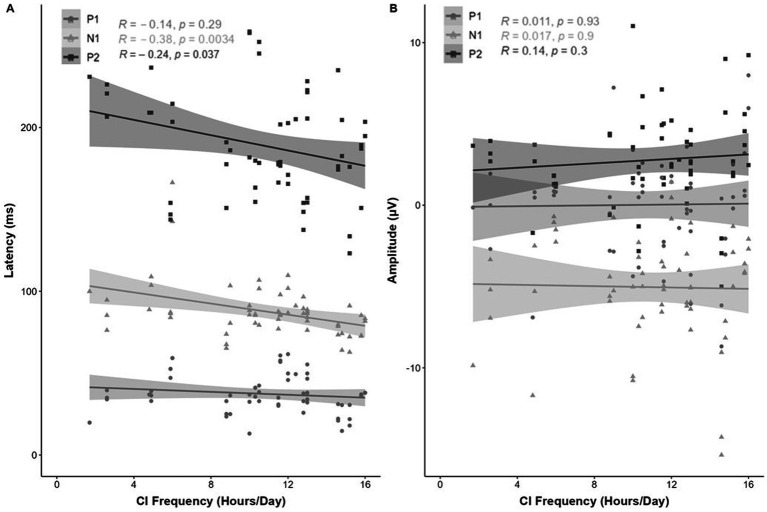
Correlation (R^2^) between eCAEP P1-N1-P2 response (**A**; latency [ms] and **B**; amplitude [μV]) and CI usage frequency (average daily CI use [hours/day]). Panel **(A)** shows a weak negative correlation between CI frequency and eCAEP response latency (ms) for N1 [*r*(69) = −0.38, *p* = 0.0034] and P2 [*r*(68) = −0.24, *p* = 0.037]. No correlation with CI frequency was observed for the P1 latency (ms). Panel **(B)** shows no correlation between CI frequency and the amplitude (μV) of the P1-N1-P2 response components.

Overall, no significant difference was observed between the eCAEP response (P1, N1, P2, inter-peak and peak-peak) latency (ms) or amplitude (μV) across the apical, medial and basal electrode contacts as detailed in Table 2. A significant association was found between eCAEP response latency (ms) and CI usage frequency. Specifically, a weak negative correlation between CI usage frequency and eCAEP response latency (ms) for N1 [*r*(69) = −0.38, *p* = 0.0034] and P2 [*r*(68) = −0.24, *p* = 0.037] was shown (Figure 5A). No correlation with CI usage frequency was observed for the latency (ms) of P1 (Figure 5A) or amplitude (μV) of the P1-N1-P2 response components (Figure 5B).

**Figure 6:**
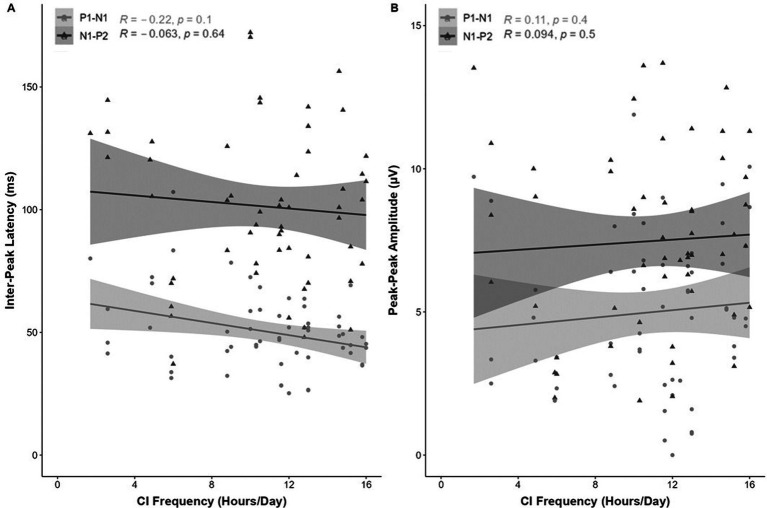
Correlation (R^2^) between eCAEP P1-N1-P2 response (**A**; inter-peak latency [ms] and **B**; peak-peak amplitude [μV]) and CI usage frequency (average daily CI use [hours/day]). No correlation was observed between CI frequency and the **(A)** inter-peak latency (ms) or **(B)** peak-peak amplitude (μV) for the P1-N1-P2 response components.

No correlation was observed between CI usage frequency and the inter-peak latency (ms) or peak-peak amplitude (μV) for the P1-N1-P2 response components (Figure 6).

**Figure 7:**
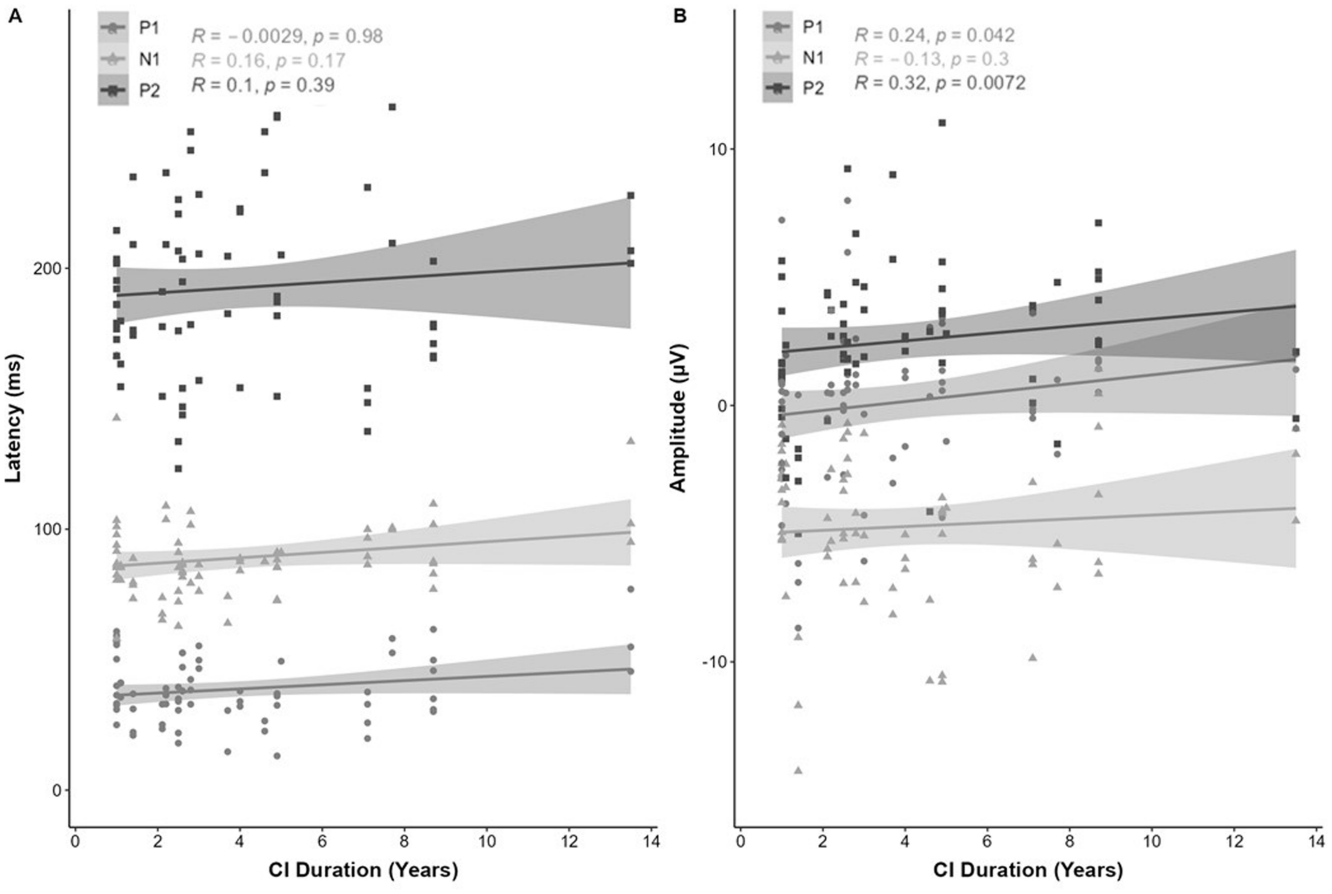
Correlation (R^2^) between eCAEP P1-N1-P2 response (**A**; latency [ms] and **B**; amplitude [μV]) and CI duration (total CI use [years]). Panel **(A)** shows no correlation between CI duration and the latency (ms) of the P1-N1-P2 response components. Panel **(B)** shows a moderate positive correlation between eCAEP response amplitude (μV) for P2 [*r*(68) = −0.32, *p* = 0.0072]. No correlation was observed for the P1 or N1 amplitude (μV).

In addition, a significant association was found between eCAEP response amplitude (μV) and CI duration (total CI use [years]), shown by a moderate positive correlation for P2 [*r*(68) = −0.32, *p* = 0.0072; Figure 7B]. No correlation was observed between CI duration and the latency (ms) of the P1-N1-P2 components (Figure 7A), or the amplitude (μV) of the P1 or N1 (Figure 7B).

**Figure 8:**
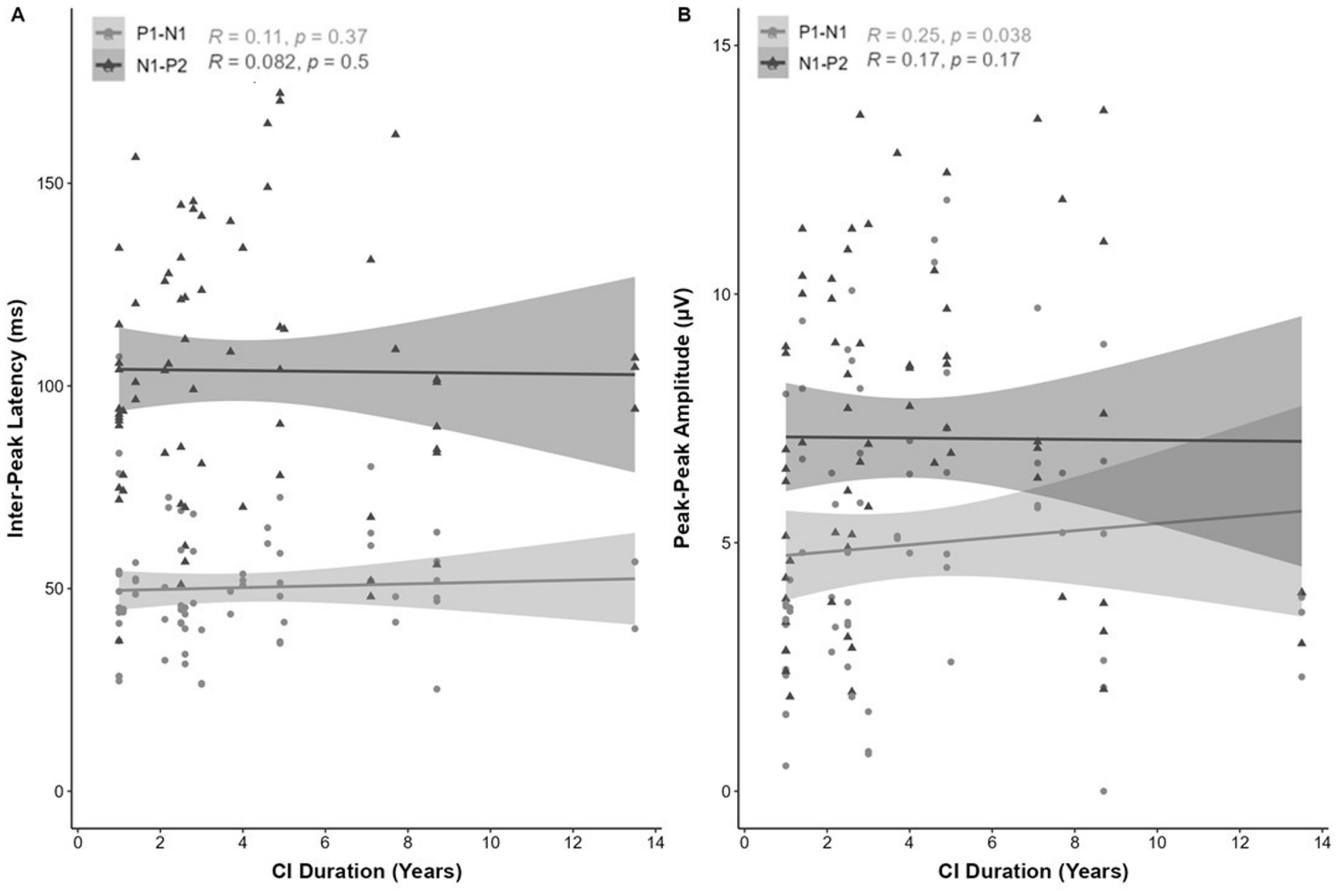
Correlation (R^2^) between eCAEP P1-N1-P2 response (**A**; inter-peak latency [ms] and **B**; peak-peak amplitude [μV]) and CI duration (total CI use [years]). Panel **(A)** shows no correlation between CI duration and the inter-peak latency (ms) of the P1-N1-P2 response components. Panel **(B)** shows a weak positive correlation between eCAEP response peak-peak amplitude (μV) for P1-N1 [*r*(68) = 0.25, *p* = 0.038]. No correlation was observed for the N1-P2 peak-peak amplitude (μV).

A weak positive correlation was shown between CI duration and eCAEP response peak-peak amplitude (μV) for P1-N1 [*r*(68) = 0.25, *p* = 0.038; Figure 8B]. No correlation was observed for the peak-peak N1-P2 (Figure 8B) amplitude (μV) or the inter-peak latency (ms) of the P1-N1-P2 response components (Figure 8A).

Table 4 presents the relationship between CI usage frequency (average daily CI use [hours/day]) and CI duration (total years of CI use). A very strong negative relationship between CI usage frequency and eCAEP response latency (ms) for the N1 (*R*^2^ = −1.76 ± 0.55, *p* = 0.002), P2 (*R*^2^ = −2.41 ± 1.11, *p* = 0.036) and P1-N1 inter-peak (*R*^2^ = −1.32 ± 0.55, *p* = 0.015) latency (ms) components was observed. Moreover, a strong positive relationship was shown between CI duration and eCAEP response amplitude (μV) for P2 (*R*^2^ = 0.518 ± 0.86, *p* = 0.008) and N1-P2 peak-peak (*R*^2^ = 0.63 ± 0.23, *p* = 0.009) amplitude (μV) components.

**Table 4:** eCAEP response linear mixed model analysis for CI usage frequency (average daily CI use [hours/day]) and CI duration (total years of CI use).

		Regression coefficient (*R*^2^)	Std Error	*t*-value	*p*-value	Sig
CI frequency (Hours/Day)						
P1	*Latency (ms)*	−0.4068	0.3878	−1.049	0.267	
	*Amplitude (μV)*	−0.01022	0.11042	−0.093	0.927	
N1	*Latency (ms)*	−1.756	0.553	−3.175	0.002	**
	*Amplitude (μV)*	−0.02044	0.11244	0.8565	0.8565	
P2	*Latency (ms)*	−2.4118	1.1165	−2.160	0.0356	**
	*Amplitude (μV)*	0.01939	0.09848	0.197	0.84467	
P1-N1	*Inter-Peak Latency (ms)*	−1.3168	0.5254	−2.506	0.0152	**
	*Peak-Peak Amplitude (μV)*	0.05183	0.09773	0.530	0.59803	
N1-P2	*Inter-Peak Latency (ms)*	−0.5123	1.1095	−0.462	0.546	
	*Peak-Peak Amplitude (μV)*	0.03153	0.12432	0.254	0.80083	
CI duration (Years)						
P1	*Latency (ms)*	−1.4083	0.7438	−1.893	0.0641	
	*Amplitude (μV)*	0.26468	0.21207	1.248	0.218	
N1	*Latency (ms)*	−0.2403	1.0438	−0.230	0.81885	
	*Amplitude (μV)*	−0.14726	0.20930	−0.704	0.4850	
P2	*Latency (ms)*	−0.2081	2.1251	−0.098	0.9224	
	*Amplitude (μV)*	0.51777	0.18744	2.762	0.00801	**
P1-N1	*Inter-Peak Latency (ms)*	0.8139	0.8580	0.3470	0.3470	
	*Peak-Peak Amplitude (μV)*	0.13444	0.15766	0.853	0.39757	
N1-P2	*Inter-Peak Latency (ms)*	−1.5828	1.8219	−0.869	0.389	
	*Peak-Peak Amplitude (μV)*	0.63004	0.23340	2.699	0.00951	**

## Discussion

This study examined the effect of CI device usage metrics on post-operative outcomes in unilateral CI recipients. The primary objective was to investigate the relationship between CI usage frequency (average daily CI use) and duration (total years of CI use) on eCAEP response peak latency (ms) and amplitude (μV), with the accompanying hypotheses that continuous and consistent daily device use would promote the development of cortical responses, thus correlating with eCAEP response metrics (amplitude [μV] and latency [ms]). Our findings supported our hypothesis that most participants (65.85%) who initially lacked acoustic CAEP responses post-surgery developed eCAEP responses after consistent CI use and increased CI experience. The remaining 34.15% exhibited an absent eCAEP response for at least one electrode contact. These findings suggest a plasticity of the auditory cortex and its ability to adapt over time. Although longitudinal evidence regarding the development of CAEP responses in adult CI users is limited, analogous research conducted in pediatric CI users has shown similar results. Specifically, the proportion of present CAEPs has been observed to significantly increase with duration of CI use (Kosaner et al., 2018). Hence, further investigation in adult populations is warranted to ascertain whether individuals lacking eCAEP responses to at least one electrode contact would exhibit the development of these responses with increased CI usage frequency over time.

The distribution of eCAEP responses across different cochlear regions was uneven, with the basal electrode contact showing the lowest rate of present responses. Specifically, for both the apical and medial electrode contacts 26 participants (63.4%) had eCAEP responses, while only 19 participants (46.3%) showed a present eCAEP response for the basal electrode contact. These findings are in line with Távora-Vieira et al. (2021), who demonstrated that the eCAEP P1–N1–P2 complex was most visible when stimulating the apical and medial electrode contacts at MCL and less visible when stimulating the basal electrode contact. Similarly, this is consistent with Liebscher et al. (2018), who demonstrated that CAEPs obtained in response to electrical stimulations at the basal area in the cochlea are less pronounced than those obtained in response to stimulation of apical and medial areas (Liebscher et al., 2018) speculates that this may be attributed, in part, to longer periods of auditory deprivation, on the basis that profound hearing loss usually manifests in the high-frequencies and, therefore, may result in an increased neural decline in the basal part of the cochlea compared to more apical areas. Moreover, the observed differences in CAEP amplitudes across the cochlea are reflected at the peripheral level. As demonstrated by Wesarg et al. (2010), electrically-evoked compound action potential thresholds increase from the apical to the basal regions. This trend is also evident in psychophysical loudness profiles (behavioral thresholds and comfort levels) as shown by Botros and Psarros (2010). Hence, lower stimulation levels are required in apical regions for equivalent loudness perception compared to basal regions, suggesting potential differences in neural density or function.

### Device use: CI usage frequency and duration

The results indicated eCAEP latency was affected by the frequency of average daily CI use (hours/day), whereby reduced CI usage frequency correlated with delayed N1 and P2 eCAEP latency (ms). Given previous research findings from our center, which demonstrated a correlation between the presence of the P1-N1-P2 complex response and higher speech perception scores in CI users (Távora-Vieira et al., 2022a; Távora-Vieira et al., 2022b), it is reasonable to suggest that increased CI usage frequency could lead to enhanced speech perception outcomes. This aligns with recent research by DeFreese et al. (2023), investigating the impact of device usage on post-operative speech recognition outcomes. DeFreese et al. (2023) found that the amount of daily device use significantly predicted approximately 20% of the variance in post-operative CI-assisted speech recognition outcomes. Similarly, Holder and Gifford (2021) demonstrated that increased daily CI use results in improved speech recognition via improved spectral processing. These findings, in conjunction with the present study, suggest that increased CI usage frequency results in improved auditory perception and speech recognition scores for adults. The collective evidence from these studies, in conjunction with our findings, is important as it highlights the importance of CI use as a factor in rehabilitating auditory perception and speech recognition in adult CI users.

### Participant factors

This research did not reveal any difference in eCAEP responses based on participants’ age at implantation, sex, or the nature and etiology of hearing loss onset. This lack of correlation suggests that the observed improvements in eCAEP responses with increased CI use are not limited by these demographic or clinical factors. It is important to acknowledge that the presence of eCAEP responses means that the speech signal is available for processing (Chang et al., 2012; Kelly et al., 2005; Abbas and Brown, 2015). However, previous research indicates that age-related synchrony differences, as well as declines in temporal resolution and duration discrimination, may impair temporal acuity of sound processing in older CI recipients (Harris and Dubno, 2017; Picton, 2013; Harris et al., 2012). The age-related decline in auditory temporal processing is reflected in the neural populations generating N1 and P2 responses, demonstrated by prolonged N1 latencies and reduced P2 amplitudes in older adults (Ostroff et al., 2003). Although older participants were more likely to have non-detectable eCAEP responses, there was no correlation between age at testing and CI usage frequency, suggesting that CI use remains consistent across age groups. Therefore, future research should be conducted in older adults to investigate the correlation between eCAEP responses and functional outcomes, particularly in the presence of background noise. Similarly, no association between the eCAEP groups and nature and etiology of hearing loss was shown, consistent with the findings published by Tavora-Vieira and Ffoulkes (2023).

### Device factors

Previous studies have indicated that evoked-potential recordings may be contaminated by CI stimulation artifacts (Gilley et al., 2006; Alemi et al., 2021). No correlation was found between eCAEP latency (ms) and amplitude (μV) and the MCL required to elicit the eCAEP response, which is consistent with the findings of Tavora-Vieira and Ffoulkes (2023). Similarly, no association was found between eCAEP response groups and device-specific factors, such as the type of implant, electrode array, magnet, audio processor, side of implantation, or stimulation mode (biphasic or triphasic). This may be due to the homogeneity of the devices used in the study; thus, future studies could benefit from including a broader range of devices to examine whether our findings hold across different CI technologies.

### Limitations

The current investigation included only participants who initially lacked CAEPs in response to acoustic stimuli. It is crucial to acknowledge the distinction between acoustically-evoked and electrically-evoked CAEP responses. However, research by Tavora-Vieira and Ffoulkes (2023) has shown a reliable correlation between these two types of stimuli in eliciting CAEP responses. Hence, it is reasonable to expect that the participants with present eCAEP responses would also have shown present aCAEP responses.

Furthermore, while the factors investigated in the present study are related to CI outcomes, they are not exhaustive and other factors warrant investigation in future research. As such, examining additional variables linked to the variability outcomes with a CI may elucidate the underlying causes for the small subgroup of users who have an unexplained absence of eCAEP responses.

## Conclusion

The present findings demonstrate the influence of CI usage frequency and duration on the presence eCAEP responses. This study suggests that greater CI duration may facilitate the development of eCAEP responses in participants who previously exhibited non-detectable, or absent, acoustic CAEP responses. Furthermore, this may suggest a potential for cortical plasticity and adaptation with consistent CI use over time. In addition, recognizing the impact of increased CI usage frequency on neural responses enhances our understanding of the importance of consistent daily CI use, which offers the potential to predict variation among CI users post-implantation. Overall, these findings may contribute to advancing the standard of personalized care in auditory rehabilitation using specific CI usage metrics to address the variability between CI users and improve post-operative outcomes.

## Data Availability

The raw data supporting the conclusions of this article will be made available by the authors, without undue reservation.
